# Einfluss eines dermatologischen Rehabilitationsprogramms auf das kardiovaskuläre Risiko bei Patienten mit Psoriasis

**DOI:** 10.1111/ddg.15585_g

**Published:** 2025-02-06

**Authors:** Jomana Al Attar, Sophia von Martial, Kaija Troost, Tobias Neumeister, Jan Ehrchen, Kerstin Steinbrink, Jochen Muke, Athanasios Tsianakas

**Affiliations:** ^1^ Klinik für Dermatologie, Fachklinik Bad Bentheim; ^2^ Medizinische Universität Münster; ^3^ Abteilung für Psychologie, Fachklinik Bad Bentheim; ^4^ Klinik für Dermatologie, Universitätsklinikum Münster; ^5^ Klinik für Kardiologie, Fachklinik Bad Bentheim, Deutschland

**Keywords:** Kardiovaskuläres Risiko, kardiorespiratorische fitness, Cardiorespiratory fitness, Cardiovaskular risk, Psoriasis, Rehabilitation, Steep Ramp Test

## Abstract

**Hintergrund und Ziele:**

Psoriasis vulgaris ist eine chronisch entzündliche Hauterkrankung, die mit zahlreichen kardiovaskulären Begleiterkrankungen und letztlich erhöhter Sterblichkeit einhergehen kann. Dermatologische Rehabilitationsprogramme sind neben der klassischen ambulanten Versorgung oder der akutstationären Behandlung eine zusätzliche therapeutische Option für Patienten mit Psoriasis. Diese Studie zielte darauf ab, die Auswirkungen einer dermatologischen Rehabilitation auf kardiovaskuläre Risikofaktoren, kardiorespiratorische Fitness und Lebensqualität in der Klinik für Dermatologie und Allergologie des Medizinischen Rehabilitationszentrums Bad Bentheim, Deutschland, zu untersuchen.

**Patienten und Methodik:**

Diese prospektive Studie umfasste 105 Patienten (Alter > 18) mit bekannter Psoriasis und/oder Psoriasis (pustulosa) palmoplantaris, die sich einem dreiwöchigen Rehabilitationsprogramm unterzogen. Verschiedene patientenbezogene Ergebnisse, einschließlich *Dermatologic Life and Quality Index* (DLQI), *Patient Global Assessment (PtGA)*, körperliche Aktivität, Pruritus sowie Nikotin‐ und Alkoholkonsumanamnese wurden erfasst. Darüber hinaus wurden der Body‐Mass‐Index (BMI) und die körperliche Fitness bewertet. Die Studienparameter wurden bei Aufnahme, Entlassung vor Ort und nach 3 und 6 Monaten telefonisch erhoben.

**Ergebnisse:**

Signifikante Verbesserungen der kardiorespiratorischen Fitness (p < 0,001), des Body‐Mass‐Index (p < 0,001), der Lebensqualität (p < 0,001), der subjektiven Einschätzung der Krankheitsaktivität durch den Patienten (p < 0,001) sowie des *Psoriasis Area and Severity Index* (PASI) (p < 0,001) wurden festgestellt.

**Schlussfolgerungen:**

Die Ergebnisse unterstreichen die Bedeutung eines Rehabilitationsprogramms für Patienten mit Psoriasis aufgrund seiner positiven und nachhaltigen Auswirkungen auf kardiovaskuläre Risikofaktoren.

## EINLEITUNG

Psoriasis ist eine chronisch entzündliche Hauterkrankung, die mit mehreren Begleiterkrankungen und Risikofaktoren wie Adipositas, Psoriasisarthritis, Lipidstoffwechselstörungen, kardiovaskulären Erkrankungen, Typ‐2‐Diabetes mellitus, Depressionen, Stress und Alkoholmissbrauch verbunden ist.^1^, ^2^, ^3^, ^4^ Sie ist eine der häufigsten entzündlichen Hauterkrankungen in Deutschland mit einer Prävalenz von etwa 2–3%.^5^

Psoriasis ist bekannt für ihre multifaktorielle Genese, die genetische, immunologische und umweltbedingte Faktoren kombiniert. Die genetische Veranlagung für Psoriasis, die auf den Chromosomen 1, 3, 6 und 19 zu finden ist, führt nicht immer zur Ausprägung des Phänotyps mit seinen charakteristischen Hautläsionen.^6^ Es gibt jedoch gut bekannte Auslösefaktoren, die zur Manifestation oder Verschlimmerung der Psoriasis führen, wie Adipositas (*Body‐Mass‐Index* [BMI] ≥ 25), Alkoholmissbrauch, Rauchen und Stress.^7^, ^8^, ^9^, ^10^, ^11^

Frühere Studien haben eine enge Korrelation zwischen kardiovaskulären Erkrankungen und Psoriasis aufgezeigt.^12^, ^13^, ^14^, ^15^ Zu diesen kardiovaskulären Erkrankungen gehört die Atherosklerose, die zu einer koronaren Herzkrankheit und Schlaganfällen führen kann, beide sind mit einer hohen Sterblichkeit verbunden.^16^ Vorsorgeuntersuchungen für kardiovaskuläre Risikofaktoren können von den konsultierenden Dermatologen durchgeführt werden und umfassen eine Blutuntersuchung auf Cholesterin (einschließlich Low‐Density [LDL]‐ und High‐Density‐Lipoprotein [HDL]‐Cholesterin), Triglyzeride (nüchtern), Glukose‐ und das glykosylierte Hämoglobin (HbA1c).^17^, ^18^

Zusätzlich ist die Psoriasis mit einer verminderten Lebensqualität verbunden.^19^ Hautläsionen, insbesondere an sichtbaren Körperstellen, stellen für die Patienten aufgrund der Stigmatisierung eine enorme Belastung dar.^20^ Die zeitaufwendige Pflege der Haut, regelmäßige ambulante Arztbesuche sowie stationäre Behandlungen führen zu einer eingeschränkten Teilnahme am täglichen Leben. Darüber hinaus leiden etwa 85% der Psoriasispatienten unter Pruritus, was zu einer noch größeren Beeinträchtigung des Lebens führt, da der Pruritus Schlafstörungen, Konzentrationsschwierigkeiten und eine weitere Stigmatisierung verursacht.^21^

Um Entzündungen zu reduzieren und die Lebensqualität zu verbessern, wurden im Laufe der Jahre verschiedene therapeutische Optionen entwickelt, die von topischen bis zu systemischen Therapien reichen. Dazu gehören entzündungshemmende topische Behandlungen wie Vitamin‐D3‐Analoga, Kortikosteroide, (Balneo‐)Phototherapie (Schmalspektrum‐UVB [UVB‐311]/Ultraviolett A [UVA]), sowie konventionelle systemische Therapien und Biologika.^22^, ^23^ Das Behandlungsziel ist es, akute Hautentzündungen zu reduzieren und asymptomatische Intervalle bei Patienten mit Psoriasis zu verlängern. Bislang gibt es jedoch aufgrund des genetischen Hintergrundes keine endgültige Heilung.^6^

Die erfolgreiche Behandlung von Psoriasis wird oft durch eine interdisziplinäre Zusammenarbeit mehrerer medizinischer Fachrichtungen erreicht.^24^ Idealerweise wird das therapeutische Management von spezialisierten Dermatologen organisiert und von Rheumatologen und Kardiologen sowie Psychologen unterstützt, um potenzielle Risikofaktoren abzudecken.^25^ In der Klinik für Dermatologie und Allergologie der Fachklinik Bad Bentheim steht dieser interdisziplinäre Ansatz im Fokus, wobei die Abteilungen für Dermatologie, Kardiologie und Psychologie fest eingebunden sind. Das dermatologische Rehabilitationsprogramm für Psoriasispatienten umfasst ein erweitertes Programm einschließlich (Balneo‐)Phototherapie, intensiver topischer Therapie sowie systemischer Therapien. Diese reichen von konventionellen Therapeutika wie Fumarsäureestern, Methotrexat, Apremilast bis hin zu Biologika wie Tumornekrosefaktor (TNF)‐α‐Inhibitoren, Interleukin (IL)‐23‐Inhibitoren bis zu IL‐17‐Inhibitoren. Darüber hinaus absolvieren die Patienten ein personalisiertes Sportprogramm, das Physiotherapie, Ergotherapie, Wassergymnastik, kardiovaskuläres Training und viele andere Aktivitäten sowie eine individuelle Ernährungsberatung und Entspannungsübungen umfasst. In Zusammenarbeit mit der Abteilung für Psychologie erhalten die Patienten bei Bedarf psychologisches Coaching sowie Evaluationstests. Das Ausleseverfahren auf kardiovaskuläre Erkrankungen und deren Behandlung gemäß den aktuellen Leitlinien der *European Society of Cardiology* (ESC) sind ebenfalls Teil des Patientenmanagements im Rehabilitationsprogramm.^26^

Bis heute wurde jedoch der Einfluss eines dermatologischen Rehabilitationsprogramms auf kardiovaskuläre Risikofaktoren bei Psoriasispatienten sowie die Nachhaltigkeit der therapeutischen Effekte noch nicht evaluiert. Ziel dieser Studie war es, die Auswirkungen eines dermatologischen Rehabilitationsprogramms auf kardiovaskuläre Risikofaktoren, kardiorespiratorische Fitness, Lebensqualität und psychische Belastung von Psoriasispatienten zu messen.

## PATIENTEN UND METHODIK

### Studienaufbau

Diese Wissenschafts‐initiierte, prospektive Studie zur Evaluierung der Auswirkungen einer dermatologischen Rehabilitation auf kardiovaskuläre Risiken, Lebensqualität und psychische Belastung bei Patienten mit Psoriasis wurde in der Fachklinik Bad Bentheim durchgeführt, einer deutschen dermatologischen Klinik, die auf die stationäre Rehabilitation von dermatologischen Patienten spezialisiert ist. Die Studie wurde vom zuständigen Ethikkomitee (Ärztekammer Niedersachsen, Niedersachsen, Deutschland) genehmigt und im Deutschen Register Klinischer Studien (DRKS00033195) registriert. Wichtige Einschlusskriterien waren Psoriasis vulgaris und/oder Psoriasis (pustulosa) palmoplantaris, die Verpflichtung zu einem dreiwöchigen Rehabilitationsprogramm in unserer Klinik und ein Alter von ≥ 18 Jahren. Die teilnahmeberechtigten Patienten wurden über einen Zeitraum von 6 Monaten rekrutiert.

**ABBILDUNG 1:**
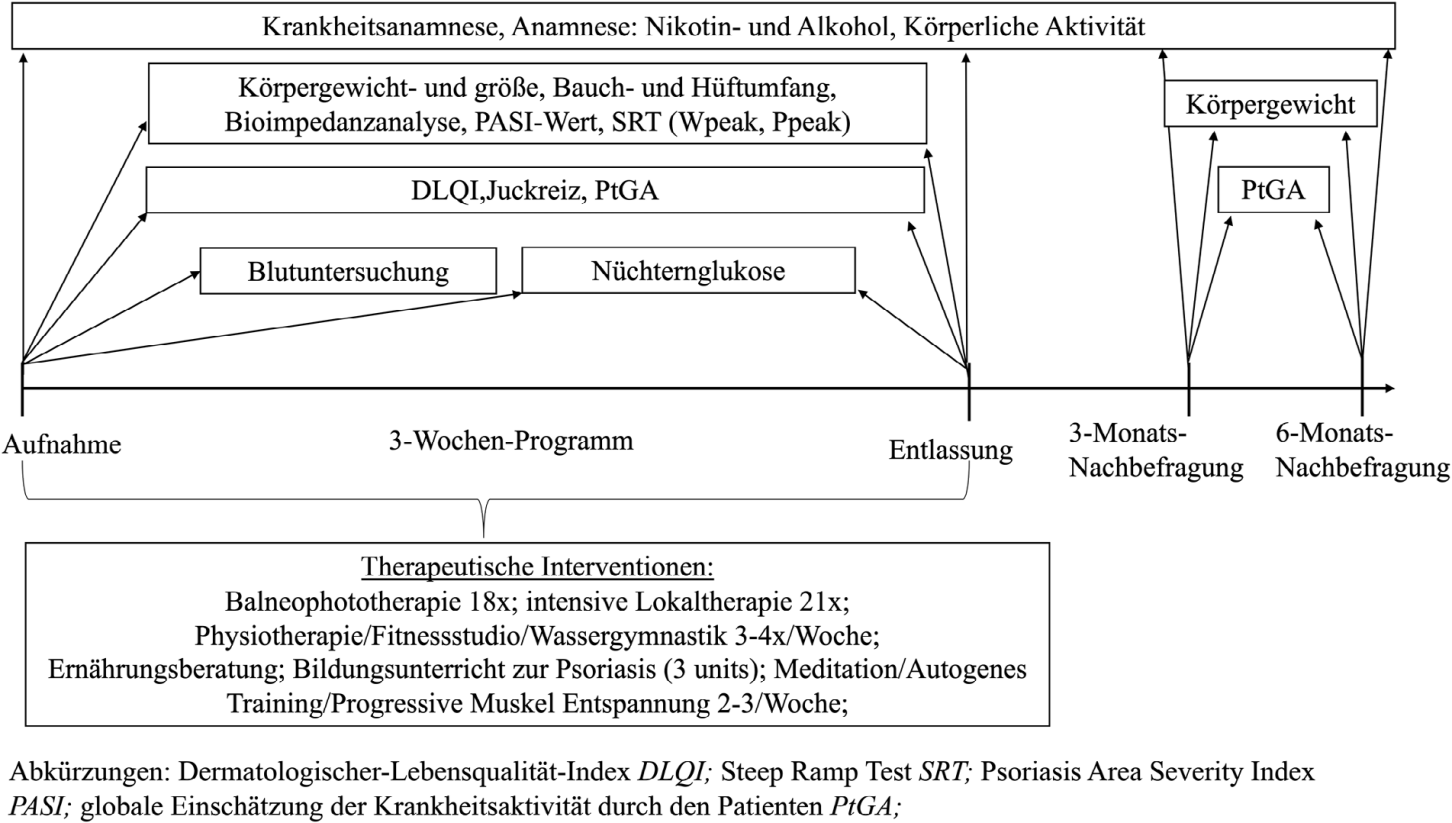
Übersicht des Studienaufbaus mit seinen spezifischen Parametern, Fragebögen und therapeutischen Interventionen.

Nach Erteilung der mündlichen und schriftlichen Einwilligung wurden die Patienten von dem behandelnden Dermatologen während des Aufnahmebesuchs für die Studie eingeschrieben. Eine umfassende Anamnese, die das Erstdiagnosedatum, frühere Behandlungen und Begleiterkrankungen sowie Risikofaktoren enthielt, wurden erhoben. Abbildung 1 bietet einen Überblick über alle Parameter und Fragebögen sowie den jeweiligen Erhebungszeitpunkt. Die Studienvisiten nach 3 und 6 Monaten wurden telefonisch durchgeführt. Eine Reihe objektiver Parameter, wie Blutdruck, Ruheherzfrequenz, Bauch‐ und Hüftumfang sowie Körpergröße und Gewicht, wurden gemessen. Der BMI wurde anschließend berechnet, indem die Körpermasse durch das Quadrat der Körpergröße dividiert wird (Angabe in kg/m^2^).^27^

Bei der Aufnahme wurde eine umfassende Laboruntersuchung durchgeführt, die ein vollständiges Blutbild, Nüchternblutzucker und Lipide, HbA1c sowie Herzmarker wie NT‐proBNP umfasste, um kardiovaskuläre Risikofaktoren zu überprüfen. Die Nüchternblutzucker‐ Werte wurden bei der Entlassung erneut gemessen.

### Bewertungsinstrumente

Zur Objektivierung der Schwere der Psoriasis wurde der *Psoriasis Area and Severity Index* (PASI) vom behandelnden Dermatologen erhoben. Er bewertet die Schwere der Läsionen durch die Beurteilung von Erythem, Infiltration und Schuppung, kombiniert mit dem Ausmaß der betroffenen Bereiche. Dies umfasst Kopf und Hals, Rumpf, Arme und Beine, wobei der Bereich von 0 (keine Erkrankung) bis 72 (maximale Erkrankung) reicht. Der PASI wurde bei der Aufnahme und bei der Entlassung gemessen.^28^

Patientenbezogene Ergebnisse zur Messung der Lebensqualität und der subjektiven Krankheitsaktivität umfassten den *Dermatologic Life and Quality Index* (DLQI) sowie die Einschätzung der Krankheitsaktivität durch den Patienten (Patient Global Assessment; PtGA). Der DLQI bewertet die Auswirkungen der Hauterkrankungen und deren Therapie auf den psychologischen Zustand, die täglichen Aktivitäten sowie die berufliche und soziale Teilhabe der Patienten. Der DLQI‐Summenwert reicht von keiner Beeinträchtigung (Summenwert 0) bis zur größten denkbaren Beeinträchtigung (Summenwert 30).^29^ Die PtGA zeigt die subjektive Einschätzung der Krankheitsschwere und reicht von 0 (keine Psoriasis) bis 5 (schwere Psoriasis).^30^ Um die durchschnittliche Intensität des Juckreizes der letzten 24 Stunden zu messen, wurde eine numerische Bewertungsskala verwendet, die von 0 (kein Juckreiz) bis 10 (schlimmster vorstellbarer Juckreiz) reicht.^31^ Zusätzlich wurde ein selbstentwickelter Fragebogen zur Einschätzung der körperlichen Fitness verwendet, der die körperliche Aktivität der Patienten pro Woche schätzte. Der Fragebogen wurde auf Basis des *International Physical Activity Questionnaire* entwickelt.^32^ Die Patienten wurden gebeten, die durchschnittliche Anzahl an Stunden (von 0 bis 13) und Tagen (von 0 bis 7) anzugeben, die sie pro Woche für körperliche Aktivität aufwendeten. Die Nikotin‐ und Alkoholkonsumanamnese wurde durch einen selbstentwickelten Fragebogen erfasst, der auf zwei Publikationen basierte.^33^, ^34^ Die Patienten wurden gebeten, die Dauer (in Jahren) sowie die Menge an Zigaretten (pro Tag) und Alkoholkonsum (Einheiten pro Woche) anzugeben.

Die kardiopulmonale Fitness wurde durch den *Steep Ramp Test* (SRT) gemessen, bei dem die Spitzenleistung in Watt (Wpeak) und die maximale Herzfrequenz in Schlägen pro Minute (Ppeak) bei der Aufnahme und Entlassung verglichen wurden. Jeder Studienpatient führte die Tests auf einem Ergometer durch. Zu Beginn wurde die Wattzahl alle 10 Sekunden um 25 Watt erhöht, wobei die Patienten angewiesen wurden, eine Trittfrequenz von 55 bis 65 Umdrehungen pro Minute beizubehalten. Die Herzfrequenz wurde zu Beginn des Tests (Ruheherzfrequenz) und nach der Spitzenleistung (Ppeak) gemessen.^35^

Das spezielle dreiwöchige Rehabilitationsprogramm umfasste verschiedene körperliche Aktivitäten, aus denen jeder Studienteilnehmer mindestens eine oder mehrere Aktivitäten in seinen täglichen Plan aufnehmen konnte. Die Teilnehmer konnten aus Fitnesskursen, Wassergymnastik, Physiotherapie und Nordic Walking wählen. Sie waren auch verpflichtet, mindestens eine Sitzung der Ernährungsberatung sowie drei Bildungsstunden zum Thema Psoriasis zu besuchen. Darüber hinaus konnten die Patienten optionale Entspannungsübungen (Qigong, Meditation, progressive Muskelentspannung, autogenes Training) wählen. Zusätzlich beinhaltete die dermatologische Behandlung eine tägliche Balneophototherapie (außer sonntags) sowie eine intensive topische Therapie.

### Statistische Methoden

Die statistische Analyse wurde mit R (Version 3.6.1), einer Software für statistische Berechnungen und Grafiken, durchgeführt.^36^ Die Eigenschaften der Patienten wurden mit deskriptiver Statistik analysiert, die Mittelwert, Standardabweichung und Median angibt. Ein abhängiger t‐Test mit gepaarten Stichproben wurde verwendet, um Mittelwert und Median von PASI, Körpergewicht, Nüchternblutzucker, Wpeak, Ppeak, DLQI und Juckreiz bei Aufnahme und Entlassung zu vergleichen. Zusätzlich wurde die Analyse der Varianz (ANOVA) verwendet, um Mittelwert und Median von PtGA, Alkohol‐ und Nikotinkonsum sowie körperlicher Aktivität von Aufnahme, Entlassung bis zur Nachuntersuchung zu vergleichen. Das Signifikanzniveau wurde bei p < 0,05 angenommen. Cohen's d wurde verwendet, um den standardisierten Unterschied zwischen den signifikanten Mittelwerten anzuzeigen.^37^

## ERGEBNISSE

### Patientencharakteristika

Insgesamt nahmen 105 Patienten an der Studie teil. Demographische Daten, Komorbidität, medizinische Vorgeschichte einschließlich Krankheitsdauer und vorherige Behandlungen sowie Lipidwerte sind in Tabelle 1 dargestellt.

**TABELLE 1:** Demographische Daten, Komorbiditäten, vorherige Therapien und Blutmarker für das kardiovaskuläre Risiko.

Patientencharakteristika	
Studienpopulation, n	105
Geschlecht (weiblich/männlich), n	43/62
Alter in Jahren, Mittelwert ± SD (Bereich)	48,72 ± 11,87 (20–70)
Krankheitsdauer in Jahren, Mittelwert ± SD	19,21 ± 15,26
PASI‐Score bei Aufnahme, Mittelwert ± SD	10,73 ± 7,67
Psoriasis vulgaris, n	101
Psoriasis palmoplantaris, n	6
Psoriasis pustulosa palmoplantaris, n	8

*Komorbiditäten*	*n (%)*
Psoriasisarthritis	27 (26)
Dyslipidämie	30 (29)
Arterielle Hypertonie	43 (41)
Hyperglykämie/Diabetes mellitus Typ 2	11 (10)
Übergewicht (BMI ≥ 25,00)	28 (27)
Adipositas	52 (50)
‐ Adipositas (BMI ≥ 30,00)	42 (40)
‐ schwere Adipositas (BMI ≥ 40,00)	10 (9)
Depression	16 (15)

*Nikotinanamnese*	
Anzahl der Raucher bei Aufnahme, n (%)	39 (37)
‐ ehemalige Raucher, n (%)	33 (31,5)
‐ Packungsjahre bei Aufnahme, Mittelwert, ± SD, Median	13,79 ± 16,04, 7,5
‐ Nie geraucht, n (%)	33 (31,5)

*Alkoholkonsum*	
Regelmäßige Alkoholtrinker bei Aufnahme, n (%)	68 (65)
Menge in Einheiten (Glas/Flasche) pro Woche, Mittelwert, ± SD, Median	1,81 ± 2,99, 1

*Systemische Therapie*	*n (%)*
Frühere systemische Therapie (jede)	65 (62)
Aktuelle systemische Therapie (Zum Zeitpunkt der Aufnahme)	34 (32)
Art der aktuellen oder früheren systemischen Therapie*	*n (%)*
Konventionell (FUM, MTX, Acitretin) Apremilast	58 (55) 5 (11)
Biologika	25 (24)
‐ TNF‐α‐Inhibitoren	18 (39)
‐ Interleukin‐17‐Inhibitoren	11 (24)
‐ Interleukin‐12/23‐Inhibitoren	7 (15)
‐ Interleukin‐23‐Inhibitoren	5 (11)
Änderung der systemischen Therapie während des Aufenthalts	4 (4)
Einleitung einer neuen systemischen Therapie während des Aufenthalts	14 (12)
‐ Fumarsäureester, n	6 (43)
‐ Methotrexat, n	4 (29)
‐ Apremilast, n	1 (7)
‐ TNF‐alpha‐Inhibitoren, n	1 (7)
‐ Interleukin‐23‐Inhibitoren, n	2 (14)

*Blutmarker bei Aufnahme (n = 105)*	*Mittelwert ± SD, Median (Referenzbereich)*
Triglyzeridspiegel in mg/dl	154,71 ± 103,95, 154,71(<200)
Cholesterinspiegel in mg/dl	197,16 ± 4,98, 197,16 (<200)
HDL‐Cholesterinspiegel in mg/dl	52,09 ± 14,04, 52,09 (> 55)
LDL‐Cholesterinspiegel in mg/dl	118,90 ± 39,16, 118,90 (<160)
HbA1c in % (n = 103)	5,83 ± 0,77, 5,6 (4,8–5,9)
NT‐proBNP in ng/l (n = 104)	52,33 ± 50,93, 35,95 (<210)
***Körperliche Aktivität (n = 105)***	***Mittelwert, ± SD, Median***
Stunden pro Woche bei Aufnahme	3,67, ± 3,68, 2
Tage pro Woche bei Aufnahme	2,77, ± 2,45, 2

*Abk*.: n, Anzahl; SD, Standardabweichung; rr, Labor‐Referenzbereich; FUM, Fumarsäureester; MTX, Methotrexat; BMI, *Body‐Mass‐Index*

*Hinweis*: Zwei Patienten hatten bei Aufnahme eine Kombinationstherapie von TNF‐α‐Inhibitor und Acitretin.

* Absolute Zahlen/Prozentsätze der Patienten

Die Analyse der Blutparameter (n = 105) zeigte, dass mehr als die Hälfte der Patienten (66%, n = 69) normale Nüchtern‐Triglyzeridwerte (0–150 mg/dl) aufwiesen. Ein Drittel (34%, n = 36) hatte jedoch eine milde bis moderate Hypertriglyzeridämie (Nüchtern‐Triglyzeride > 150 mg/dl). 47% (n = 49) der Patienten hatten abnormale Cholesterinwerte (>200 mg/dl). Einundzwanzig Prozent der Studienpopulation (n = 22) wiesen abnormale HDL‐Werte (<40 mg/dl) auf und mehr als die Hälfte der Patienten (55%, n = 58) hatte erhöhte LDL‐Cholesterinwerte (> 110 mg/dl).

Um das Risiko für ventrikuläre Dysfunktion und Herzinsuffizienz in der Studienpopulation (n = 104; ein Laborfehler während der Labortests) zu messen, wurden NT‐proBNP‐Werte (in ng/l) ermittelt. Fast alle Patienten (93%, n = 97) zeigten normale NT‐proBNP‐Werte (<125 ng/l). Sieben Prozent (n = 7) zeigten Werte von 125–300 ng/l. Keiner der Teilnehmer wies NT‐proBNP‐Werte über 300 ng/l auf, was auf eine akute Herzinsuffizienz hindeuten würde.^38^

Bei der Aufnahme gaben 10% der Patienten (n = 11) an, an Diabetes mellitus zu leiden. Die Analyse des HbA1c (n = 103) zeigte jedoch, dass 35% (n = 36) der Population abnormale HbA1c‐Werte hatten, wobei 23% (n = 24) sich im prädiabetischen Zustand (HbA1c 5,7–6,5%) und 12% (n = 12) im diabetischen Zustand (HbA1c > 6,5%) befanden. Fünf Prozent (n = 5) der Population hatten sehr hohe HbA1c‐Werte > 7,5%. Mehr als die Hälfte der Patienten (65%, n = 67), hatten normale HbA1c‐Werte (<5,7%). Die Nüchtern‐Glukosewerte (n = 105) wurden bei Aufnahme und Entlassung gemessen. Der Mittelwert der Nüchtern‐Glukosewerte stieg signifikant von der Aufnahme (93,21 ± 22,69 mg/dl) bis zur Entlassung (102,29 ± 24,76 mg/dl) (p <0,001).

Die Analyse der Nikotinanamnese zeigte, dass 70% (n = 70) der Patienten zum Zeitpunkt der Aufnahme ehemalige oder aktuelle Raucher waren. Der Mittelwert der Packungsjahre (Packungen pro Tag x Jahre als Raucher) der Patienten mit Raucheranamnese betrugen 13,79 (± 16,04, Median 7,5). Bei der Aufnahme waren 37% der Patienten (n = 39) regelmäßige Raucher, im Vergleich zu 38% (n = 40) bei der Entlassung. Nach einer dreimonatigen Nachuntersuchung waren 37% (n = 39) noch immer regelmäßige Raucher, im Vergleich zu 35% (n = 37) nach einer sechsmonatigen Nachuntersuchung (p = 0,99, nicht signifikant).

Die Anamnese des Alkoholkonsums zeigte, dass 65% der Patienten bei der Aufnahme angaben, regelmäßig Alkohol zu konsumieren. Der Mittelwert der Menge an Alkohol pro Einheit/Woche (Glas/Flasche) verringerte sich signifikant von der Aufnahme (1,81 ± 2,99, Median 1) bis zur Entlassung (0,44 ± 0,76, Median 0) (p < 0,001). Nach einer dreimonatigen Nachuntersuchung stieg der Mittelwert der Menge an Alkohol pro Einheit/Woche auf 1,72 (± 2,73, Median 1) signifikant im Vergleich zur Entlassung an (p < 0,001). Bei der sechsmonatigen Nachuntersuchung änderte sich der Mittelwert des Alkoholkonsums (1,71 ± 2,16, Median 1) nicht signifikant (p = 0,9999). Der Alkoholkonsum änderte sich von der Aufnahme bis zur Nachuntersuchung nicht signifikant (p = 0,9952).

**ABBILDUNG 2:**
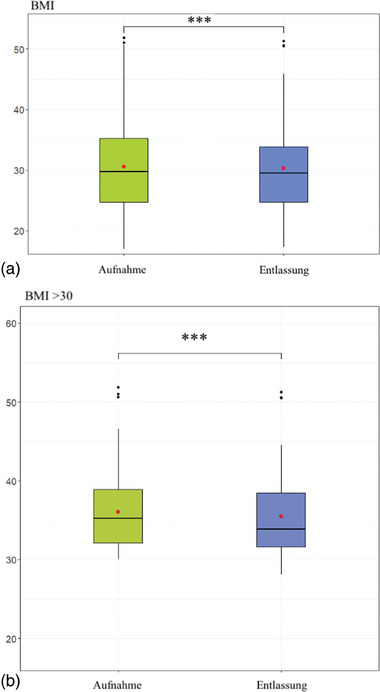
Body‐Mass‐Index (BMI) bei Aufnahme und Entlassung. Body‐Mass‐Index während des Rehabilitationsprogramms im (a) gesamten Patientenkollektiv (n = 105) und (b) adipösen Patientenkollektiv (n = 42) bei Aufnahme und Entlassung (***p < 0,001).

Der BMI im gesamten Patientenkollektiv (n = 105) und im adipösen Patientenkollektiv (BMI > 30; n = 42) bei Aufnahme und Entlassung ist in Abbildung 2a und b dargestellt. Der Mittelwert des BMI im gesamten Patientenkollektiv betrug bei der Aufnahme 30,60 (± 7,05, Median 29,9) im Vergleich zu 30,32 (± 6,79, Median 29,59) bei der Entlassung (p < 0,001). Der signifikante Rückgang wurde von den adipösen Patienten angeführt (der Mittelwert des BMI im adipösen Kollektiv betrug bei der Aufnahme 36,03 (± 5,61, Median 35,20) im Vergleich zu 35,46 (± 5,47, Median 33,87) bei der Entlassung (p < 0,001).

Hüft‐ und Taillenumfang verbesserten sich von der Aufnahme bis zur Entlassung nicht signifikant (Daten nicht gezeigt). Außerdem gab es keine signifikante Verbesserung der körperlichen Fitness von der Aufnahme bis zur Entlassung und bis zur Nachuntersuchung (Daten nicht gezeigt).

**ABBILDUNG 3:**
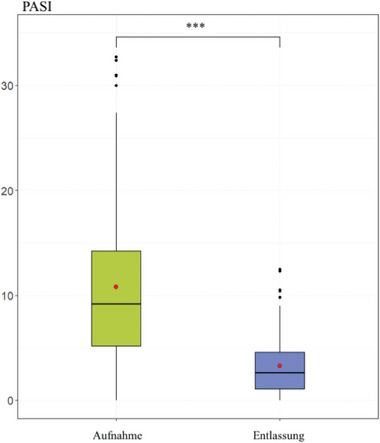
Psoriasis *Area and Severity Index* (PASI) bei Aufnahme und Entlassung. Verbesserung des PASI während der Rehabilitationsbehandlung in der Gesamtpatientengruppe (n = 105) im Vergleich der PASI‐Werte bei Aufnahme und Entlassung (***p < 0,001).

Bei der Aufnahme zeigten 53% (n = 56) der Studiengruppe eine leichte Psoriasis mit PASI‐Werten unter 10. Sechsunddreißig Prozent (n = 38) wiesen eine mittelschwere bis schwere Psoriasis mit PASI‐Werten von 10 bis 20 auf, und bei 11% der Patienten (n = 11) wurde eine schwere Psoriasis mit PASI‐Werten über 20 festgestellt. Bei der Entlassung wurden 96% (n = 101) der Studienpopulation mit leichter Psoriasis diagnostiziert (PASI‐Werte unter 10). Abbildung 3 zeigt die Verbesserung des Mittelwerts des PASIs während der Rehabilitationsbehandlung. Der Mittelwert verbesserte sich signifikant von mittelschwerer bis schwerer Psoriasis (10,73 ± 7,67) bei Aufnahme zu leichter Psoriasis (3,26 ± 2,76) bei Entlassung (p < 0,001).

### Kardiorespiratorische Fitness

**ABBILDUNG 4:**
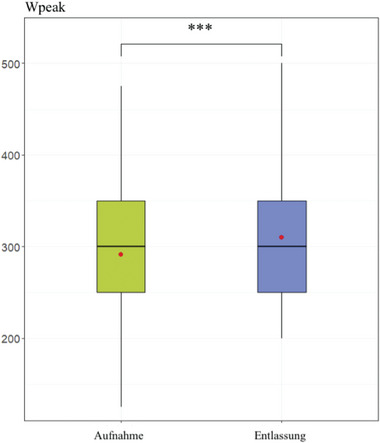
Spitzenleistung während des *Steep Ramp Test*. Mittelwert und Median Wpeak im Vergleich bei Aufnahme und Entlassung (***p < 0,001).

Der SRT wurde durchgeführt, um die kardiorespiratorische Fitness (n = 105) bei Aufnahme und Entlassung zu bewerten. Wie in Abbildung 4 dargestellt, betrug der Wpeak bei Aufnahme 291,67 (± 69,12) und stieg signifikant bei Entlassung auf 310,48 (± 71,53) an (p < 0,001). Ppeak lag bei Aufnahme bei 142,88 (± 20,84) und bei Entlassung bei 145,76 (± 21,37) (p = 0,076, nicht signifikant).

### Patientenbezogene Ergebnisse

**ABBILDUNG 5:**
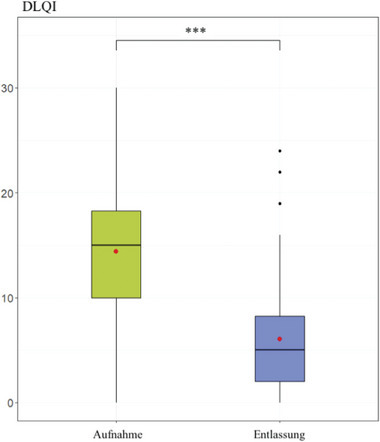
Dermatologic *Life and Quality Index* (DLQI) bei Aufnahme und Entlassung. Mittelwert und Median des DLQI im Vergleich bei Aufnahme und Entlassung (***p < 0,001).

Alle von den Patienten berichteten Ergebnisse verbesserten sich signifikant. Der DLQI bei Aufnahme zeigte einen sehr großen Einfluss (14,41 ± 6,57) der Psoriasis auf die Lebensqualität der Patienten, der sich signifikant auf einen moderaten Einfluss auf das Leben der Patienten (6,06 ± 5,23) verringerte (p < 0,001), wie in Abbildung 5 dargestellt.

**ABBILDUNG 6:**
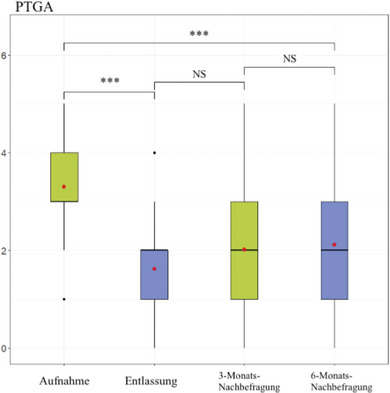
Patientenbezogene Bewertung bei Aufnahme, Entlassung und Nachuntersuchungen. Mittelwert und Median des PtGA im Vergleich bei Aufnahme, Entlassung, nach 3 und 6 Monaten (***p < 0,001; **p < 0,01; NS, nicht signifikant).

Der PtGA wurde bei Aufnahme, Entlassung und bei beiden Nachuntersuchungen gemessen, wie in Abbildung 6 gezeigt. Der Mittelwert des PtGA betrug bei Aufnahme 3,29 (± 1,09, Median 3), bei Entlassung 1,62 (± 0,81, Median 2), nach 3 Monaten 2,02 (± 1,28, Median 2) und nach 6 Monaten 2,12 (± 1,33, Median 2) (p < 0,001).

Der Juckreiz wurde bei Aufnahme als leicht bis moderat angegeben (4,58 ± 3,22), was sich signifikant auf leicht bis keinen Juckreiz bei Entlassung verringerte (1,52 ± 2,11) (p < 0,001).

## DISKUSSION

Ziel dieser prospektiven Evaluationsstudie war es, den Einfluss einer dermatologischen Rehabilitation auf kardiovaskuläre Risikofaktoren, Lebensqualität und psychische Belastung von Psoriasispatienten zu untersuchen. Die Rehabilitation hatte einen signifikanten Einfluss auf die kardiorespiratorische Fitness, den BMI, die Lebensqualität sowie auf die objektive und subjektive Krankheitsaktivität. Die Patientenmerkmale dieser Studie entsprechen denen anderer Studien, insbesondere in Bezug auf die hohe Anzahl an Begleiterkrankungen bei Psoriasis.^39^, ^40^, ^41^ Daher stellt die Kohorte eine typische Gruppe von Psoriasispatienten dar.

Die Patientenmerkmale zeigten, dass fast die Hälfte der Studienpopulation bei Aufnahme an einer mittelschweren bis schweren Psoriasis litt. 53% der Patienten hatten bei Aufnahme eine leichte Psoriasis. Die Rehabilitationsmaßnahme wird oft vom Hausarzt oder Dermatologen während eines akuten Psoriasis‐Schubes eingeleitet, aber es dauert häufig mehr als zwei bis 3 Monate bis zur Aufnahme. Dies erhöht die Wahrscheinlichkeit einer spontanen Remission, insbesondere bei saisonalen Veränderungen. Zusätzlich kann in der Zwischenzeit eine ambulante Therapie begonnen werden, die (balneo)phototherapeutische sowie systemische Behandlungsmöglichkeiten umfasst. Nach dem aktuellen Prinzip *hit hard and early* zeigten Studien bereits 2013, dass eine frühzeitige systemische Behandlung der Psoriasis große Vorteile im Hinblick auf kardiovaskuläre Begleiterkrankungen und den entzündlichen Charakter der Krankheit haben kann.^42^, ^43^ Besonders biologische Therapien verbessern die langfristigen Ergebnisse der Psoriasis erheblich.^44^ Bei Aufnahme waren 32% der Studienpopulation mit einer systemischen Therapie behandelt, wobei davon 47% mit biologischen Medikamenten behandelt wurden. Allerdings erhielten ein Drittel der Patienten bei Aufnahme keine systemische Therapie bis zum jetzigen Zeitpunkt. Zwölf Prozent der Studiengruppe begannen während des Rehabilitationsaufenthalts eine neue systemische Therapie. Dies unterstreicht die Bedeutung der dermatologischen Rehabilitation auch im Hinblick auf die Therapieoptimierung.

Hinsichtlich der Begleiterkrankungen gaben interessanterweise nur 10% der Studienpopulation bei der Aufnahmeuntersuchung an, an Diabetes mellitus zu leiden. Die Studienergebnisse zeigten jedoch im Gegenteil, dass 35% der Population abnormale HbA1c‐Werte aufwiesen. Gründe für diese Entdeckung könnten hauptsächlich ein Mangel an Vorsorgeuntersuchung durch Hausärzte (und Dermatologen) sein. Ein weiterer Grund könnte das mangelnde Wissen der Psoriasispatienten über häufige Begleiterkrankungen sein. Patienten mit erhöhtem HbA1c wurden an den Internisten überwiesen, und es wurden entsprechende Diät‐ und/oder spezifische antidiabetische Behandlungen eingeleitet. Um unentdeckten hohen Blutzucker bei Psoriasispatienten vorzubeugen, sollten Dermatologen ihre Patienten und/oder Hausärzte anweisen, regelmäßig Blutzuckertests durchzuführen. Im Idealfall sollte jeder Psoriasispatient mindestens einmal jährlich bei seinem Hausarzt Blutuntersuchungen, einschließlich Blutlipide und Blutzucker, durchführen lassen. Frühere Studien haben gezeigt, dass Diabetes mellitus mit einem höheren Risiko für die Entwicklung einer ventrikulären Dysfunktion verbunden ist, was letztlich zu einer erhöhten Sterblichkeit führen kann.^45^ Darüber hinaus kann eine verzögerte Behandlung des Diabetes aufgrund von Unkenntnis zu schwerwiegenden Folgen wie Polyneuropathie, Retinopathie oder sogar Herzinsuffizienz führen.^46^, ^47^ Dies unterstreicht die Bedeutung regelmäßiger Vorsorgeuntersuchungen auf Begleiterkrankungen bei Psoriasispatienten, die in der täglichen Praxis bei Dermatologen oft noch nicht ausreichend durchgeführt werden. Somit konnte durch diese Studie der Nutzen eines dermatologischen Rehabilitationsprogramms bei der Früherkennung und dem Aufspüren potenziell lebensbedrohlicher Begleiterkrankungen nachgewiesen werden.

Hinsichtlich der Begleiterkrankungen des Herz‐Kreislauf‐Systems zeigte die Studie, dass 7% der Population abnormale NT‐proBNP‐Werte aufwiesen, was zu einem erhöhten Risiko für die Entwicklung einer ventrikulären Dysfunktion führt. Die Studienergebnisse zeigten, dass 10% der Patienten angaben, Antikoagulanzien aufgrund von Herz‐Kreislauf‐Erkrankungen oder Thrombosen einzunehmen. Somit konnte die Studie durch Labortests keine weiteren Herz‐Kreislauf‐Erkrankungen aufdecken. Wir können folgern, dass NT‐proBNP als Vorsorgeuntersuchung nicht unbedingt empfohlen werden sollte, wenn es um die Untersuchung auf Begleiterkrankungen geht. Stattdessen sollten Ärzte andere Testverfahren bevorzugen, wie zum Beispiel das Elektrokardiogramm oder Echokardiogramm.

Die Nikotin‐ und Alkoholkonsumanamnese von der Aufnahme bis zur Nachuntersuchung zeigte keine signifikante Reduktion des Nikotin‐ oder Alkoholverbrauchs. Studien haben jedoch gezeigt, dass eine enge Korrelation zwischen der Schwere der Psoriasis und der Behandlungseffizienz mit Nikotin‐ und Alkoholkonsum besteht. Ein erhöhter Konsum von Nikotin und Alkohol kann die Behandlungsergebnisse erheblich verschlechtern und so den Heilungsprozess beeinträchtigen. Eine Querschnittsstudie von Wei et al., die 2021 in China durchgeführt wurde, zeigte, dass Raucher mindestens 1,2‐mal höhere PASI‐Werte hatten als Nichtraucher.^48^, ^49^, ^50^, ^51^ Nikotinkarenz ist eines der relevantesten Themen des letzten Jahrzehnts. Studien zeigen, dass es in der Regel 30 Versuche benötigt, um das Rauchen aufzugeben.^52^, ^53^ Die Rückfallrate beim Rauchen zeigt ähnliche wenig vielversprechende Ergebnisse, mit einer Rückfallrate von etwa 62%.^54^, ^55^ Interessanterweise ist in unserer Untersuchung ein Patient während des Rehabilitationsaufenthalts rückfällig geworden. Um dieser Tendenz entgegenzuwirken, wurden die Patienten ermutigt, an einem intensiven Nikotinkarenz‐Programm teilzunehmen. Die Teilnahme an diesem Programm war jedoch freiwillig. Nur elf von 37 Rauchern nahmen am Programm teil, was ein weiterer Grund für die nichtsignifikanten Ergebnisse sein könnte. Um die Nikotinkarenz bei Psoriasispatienten zu verbessern, könnten Nikotinkarenz‐Programme verpflichtend gemacht werden. Studien haben gezeigt, dass bereits eine kurze ärztliche Anweisung zur Nikotinkarenz die Aufhörquoten erhöhen kann,^56^, ^57^ sodass eine Karenz einfach durch eine proaktivere Ermutigung der Ärzte während der Rehabilitation erreicht werden könnte. Zusammenfassend lässt sich sagen, dass die Rehabilitation nur ein Schritt auf dem Weg zur Nikotinkarenz sein kann und nur langfristige Ergebnismessungen den tatsächlichen Nutzen des Rehabilitationsaufenthalts in Bezug auf die endgültige Nikotinkarenz zeigen können.

Im Verlauf des Rehabilitationsaufenthalts erhielten die Patienten Ernährungsberatung, Physiotherapie, Zugang zum Schwimmbad und eine Vielzahl von Bewegungsaktivitäten wie Gehtraining, Radtouren und angeleitete Übungen zum Gerätetraining. Ziel war es, die Energieaufnahme zu reduzieren, Verhaltensänderungen einzuleiten sowie den Energieverbrauch zu erhöhen, um Übergewicht erfolgreich zu bewältigen. Es gab eine signifikante Verbesserung des BMI von der Aufnahme bis zur Entlassung in der Gesamtpatientengruppe. Dies ist hauptsächlich auf die signifikante Verbesserung des BMI in der Gruppe der Adipösen (BMI > 30) zurückzuführen, was beweist, dass die signifikante Reduktion des BMI auf den Rückgang der Kohorte mit den höchsten BMI‐Werten zurückzuführen ist, die das höchste Risiko für kardiovaskuläre Ereignisse aufweisen.^58^, ^59^ Die Untersuchung belegt derzeit, dass die dermatologische Rehabilitation tatsächlich Übergewicht reduzieren und kontrollieren und somit die kardiovaskuläre Gesundheit bei Psoriasispatienten verbessern kann. Übergewicht ist eine der häufigsten Todesursachen in Europa aufgrund seiner engen Verbindung zu Herz‐Kreislauf‐Erkrankungen.^60^ Strategien zur Bekämpfung der Sterblichkeit durch Übergewicht und dessen Folgen gehören zu den größten Sorgen der Industrieländer weltweit. Wir wissen bereits, dass Psoriasispatienten ein erhöhtes Risiko für Übergewicht und Herz‐Kreislauf‐Erkrankungen haben, was die Bedeutung der Ergebnisse und der anschließenden Strategien zur Bekämpfung des Problems unterstreicht. Kürzlich wurden neuere Strategien identifiziert, die Diabetes‐Medikamente wie Glucagon‐like‐Peptide‐1‐Rezeptor (GLP1‐RA)‐Agonisten als eine der vielversprechendsten Interventionen gegen Übergewicht herausgestellt haben.^61^, ^62^, ^63^ Darüber hinaus haben GLP1‐RA‐Agonisten gezeigt, dass sie signifikant kardiovaskuläre Ereignisse und damit verbundene Risiken wie Blutdruck bei Diabetes‐ und Nicht‐Diabetes‐Patienten reduzieren können.^64^, ^65^, ^66^ Psoriasispatienten könnten erheblich von diesen Medikamenten profitieren, da Gewichtsverlust bei adipösen Patienten die Schwere und das Ausmaß der Psoriasis (PASI‐Werte) erheblich verbessert, was zu einer besseren Lebensqualität führt. Noch wichtiger ist, dass es kardiovaskuläre Risikofaktoren reduzieren und letztendlich die Sterblichkeit bei Psoriasispatienten senken kann.^67^, ^68^, ^69^

Die Anamnese zur körperlichen Aktivität zeigte von der Aufnahme über die Entlassung bis zur Nachuntersuchung keine signifikanten Änderungen. Studien haben jedoch gezeigt, dass regelmäßige körperliche Aktivität mit einem verbesserten Krankheitsverlauf, einem reduzierten kardiovaskulären Risiko und einer verbesserten Lebensqualität verbunden ist.^70^, ^71^ Um diese Ergebnisse zu verbessern, gibt es bereits einige Nachsorgebehandlungsoptionen, wie zum Beispiel das Intensivierte Rehabilitationsnachsorgeprogramm (IRENA) und das trainingsorientierte Rehabilitationsnachsorgeprogramm (T‐RENA).

Die Studie zeigte eine signifikante Verbesserung der PASI‐Werte, die mit einer erheblichen Reduzierung der Krankheitsaktivität korrelierte. Während des Programms profitierten die Patienten von einer intensiven Balneophototherapie sowie lokaler Therapie. Im ambulanten Bereich wird die Balneophototherapie normalerweise, falls überhaupt, ein‐ bis zweimal pro Woche durchgeführt, im Vergleich zu sechsmal pro Woche während des Rehabilitationsprogramms. Während des Programms haben die Patienten die Möglichkeit, ihre hauptsächliche Zeit für die Hautpflege zu nutzen, während sie im ambulanten Bereich durch Arbeit und Alltagsangelegenheiten beschäftigt sind. Darüber hinaus hatten 15% der Studiengruppe trotz Bedarf an neuer oder anderer Psoriasis‐Behandlung eine unzureichende oder gar keine systemische Therapie. Ein niedrigerer PASI‐Wert ist mit einer verbesserten Lebensqualität und einem geringeren Risiko für Depressionen verbunden.^72^, ^73^ Um die PASI‐Werte zu verbessern und damit die Lebensqualität zu erhöhen, sollten Ärzte ihre Patienten häufiger an spezialisierte Kliniken überweisen.

Die Studie zeigte eine signifikante Verbesserung des Wpeak während des SRT von der Aufnahme bis zur Entlassung. Der SRT wurde verwendet, um die kardiorespiratorische Fitness der Studienpopulation zu messen, was aufgrund der bekannten kardiovaskulären Begleiterkrankungen bei Psoriasispatienten von großer Bedeutung ist. Die Überwachung der kardiorespiratorischen Fitness ist äußerst wichtig, da ein umgekehrter Zusammenhang zwischen kardiorespiratorischer Fitness und Sterblichkeit besteht.^74^ Darüber hinaus gibt es eine Assoziation zwischen reduzierter körperlicher Fitness und Schlaganfällen sowie anderer nichttödlichen Herz‐Kreislauf‐Erkrankungen.^75^, ^76^ Die Ergebnisse zeigten, dass die kardiorespiratorische Fitness (Wpeak) während des Rehabilitationsaufenthalts signifikant verbessert wurde. Wichtig ist, dass die Daten erstmals zeigen, dass bereits ein dreiwöchiges ganzheitliches dermatologisches Rehabilitationsprogramm die kardiorespiratorische Fitness bei Psoriasispatienten signifikant erhöht hat und somit dazu beitragen kann, das kardiovaskuläre Risiko mit seinen bekannten ernsten Folgen zu verringern. Kardiologische Studien zur kardiovaskulären Fitness haben ähnliche Erfolge in einem so kurzen Zeitraum gezeigt.^77^, ^78^

Bei der Analyse patientenbezogener Daten zeigte die Studie eine signifikante Verbesserung des Juckreizes, der krankheitsbezogenen Lebensqualität und der subjektiven Krankheitsaktivität von Aufnahme bis zur Entlassung. Die Verbesserung der Lebensqualität ist ein sehr wichtiges Ziel in der langfristigen Behandlung von Psoriasispatienten, da eine enge Beziehung zwischen Depressionen und Psoriasispatienten besteht. Die Studie zeigte, dass 15% der Studienpopulation bei Aufnahme angaben, unter Depressionen zu leiden. Diese Zahl ist im Vergleich zur Allgemeinbevölkerung in Deutschland (Frauen 11,6%; Männer 8,6%; Gesamtprävalenz 10,1%) deutlich höher, basierend auf einer Querschnittsstudie des Robert Koch‐Instituts von 2017.^79^ Vergleichbare Zahlen zur Depression bei Psoriasispatienten sind ähnlich.^80^ Depressionen sind bekanntlich mit einem erhöhten Risiko für Herz‐Kreislauf‐Erkrankungen verbunden, was zu einer erhöhten Sterblichkeit führt.^81^, ^82^, ^83^ Die Untersuchung zeigte eine signifikante Verbesserung des DLQI und PtGA bei Patienten mit Psoriasis, was letztlich zu einer Reduktion der depressiven Symptome führen könnte, begleitet von einer anschließenden Reduktion des kardiovaskulären Risikos und damit der Sterblichkeit bei Psoriasispatienten.

Frühere Studien haben bereits gezeigt, dass stationäre Rehabilitationsprogramme die PASI‐Werte, den langfristigen Krankheitsverlauf und die psychologische Beeinträchtigung erheblich verbessern können.^84^, ^85^, ^86^ Diese Ergebnisse werden durch unsere Studie bestätigt. Diese Studie ist jedoch die erste, die signifikante Effekte auf kardiovaskuläre Risiken bei Psoriasispatienten zeigt, was dazu beitragen kann, größere Gesundheitsprobleme zu reduzieren.

### Schlussfolgerungen

Ein interdisziplinäres dermatologisches Rehabilitationsprogramm, wie es in der Fachklinik Bad Bentheim etabliert ist, erhöht die kardiorespiratorische Fitness signifikant, reduziert Übergewicht und verbessert die Lebensqualität bei Psoriasispatienten. Darüber hinaus führt es zu einer nachhaltig verbesserten Krankheitsaktivität für mehr als 6 Monate nach der Entlassung. Die Kombination aller Disziplinen unter einem Dach ist ein echter Gewinn für Psoriasispatienten, ambulante Dermatologen und auch für das Gesundheitssystem. Psoriasispatienten profitieren von einer umfassenden Vorsorgeuntersuchung. Die Untersuchung zeigte auffällige HbA1c‐Werte bei Patienten, die nichts von ihren Glukosestörungen wussten. Die Früherkennung von Diabetes kann teure Behandlungen für Patienten, Ärzte und Krankenkassen vermeiden. Darüber hinaus haben Patienten leichteren Zugang zu verbesserten Behandlungsstrategien, einschließlich systemischer Therapie. Zwölf Prozent der Studiengruppe erhielten während des Aufenthalts eine neue systemische Therapie. Die Ergebnisse bestätigen auch die Notwendigkeit eines interdisziplinären Ansatzes in der Behandlung von Psoriasispatienten im Hinblick auf die Vielfalt der (kardiovaskulären) Begleiterkrankungen innerhalb der Studienpopulation. Die Überweisung an ein Rehabilitationszentrum verbessert nicht nur signifikant die Versorgungsqualität der Patienten und Praxen, sondern auch erheblich die Hautläsionen und die Lebensqualität. Am wichtigsten ist, dass wir bereits nach 3 Wochen sowohl einen signifikant reduzierten BMI als auch eine signifikant erhöhte kardiovaskuläre Fitness nachweisen konnten, was potenziell die kardiovaskulären Risikofaktoren und damit die Sterblichkeit reduzieren könnte.

Aufgrund der nichtsignifikanten Ergebnisse bezüglich des Nikotin‐ und Alkoholkonsums sowie der von den Patienten berichteten körperlichen Aktivität sollte während der stationären Behandlung sowie im Nachsorgeprogramm mehr Augenmerk auf ein intensiveres Programm für körperliche Aktivität und Nikotin‐ bzw. Alkoholkarenz gelegt werden. Aufgrund der geringen Patientenzahl sollten die Ergebnisse dieser Studie mit einer größeren Patientengruppe einschließlich einer Kontrollgruppe an mehreren Studienstandorten reproduziert werden, um die Studienergebnisse zu verifizieren.

## INTERESSENKONFLIKT

Keiner.
